# Supplementary material for: Risk factors for melioidosis in Udupi District, Karnataka, India, January 2017-July 2018
Source: PLOS Glob Public Health. 2022 Dec 13;2(12):e0000865. doi: 10.1371/journal.pgph.0000865 (PMC10021467; doi:10.1371/journal.pgph.0000865)
Supplement: S1 Fig — (DOCX) [file pgph.0000865.s001.docx]

S1 Fig. Flow diagram showing selection of Melioidosis Cases, in the case control study, Udupi District, Karnataka

1:3 matched case control study, cases=19, control=56

A subset of cases (19) admitted from January 2017- July 2018 enrolled for the case control

Descriptive analysis by time, place and person (n=50)

Between 2013-18, 50 melioidosis cases admitted in hospital A

Excluded all 31 case from 2013-2016
