# Supplementary material for: Risk factors for melioidosis in Udupi District, Karnataka, India, January 2017-July 2018
Source: PLOS Glob Public Health. 2022 Dec 13;2(12):e0000865. doi: 10.1371/journal.pgph.0000865 (PMC10021467; doi:10.1371/journal.pgph.0000865)
Supplement: Attachment — Submitted filename: Response to reviewers.docx [file pgph.0000865.s002.docx]

Summary

Reviewer 1

| S. No. | Comment | Response |
| --- | --- | --- |
|  | Major | |
| 1 | Line 149: please explain why only 19 of the 50 cases were analyzed. It might be best to include a flow diagram with inclusions and exclusions for clarity. | Thank you for the suggestion. A flowchart illustrating the details of enrollment process for case control study has been added as annexure page 2 and referenced in the text at line 167. |
|  | Line 202: there is no mention of severity in the case definition, but is mentioned as a factor in the limitation sections. Please clarify. | Thank you for pointing out this omission. The sentence has been paraphrased as *hospitalized cases from a single hospital were enrolled, potentially limiting the generalizability of our findings.* Changes are at line 216-217. |
|  | It has been mentioned that melioidosis is not a notifiable condition in India. There are a number of instances in the manuscript where the term “reported” or “reporting” has been used, for example in Figure 2. For clarity are these the cases identified by the investigation or the cases sporadically reported to the NCDC? | The case plotted on Figure 2 are cases admitted to hospital A as per our case definition given in line 101-103.  For clarity the word “reported “has been deleted and replaced by “*admitted*” in figure 2. |
|  | There is no mention of additional investigations relating to the case. Did he have pneumonia? Was he bacteremic? Was a swab of the abrasion site taken, and if so was it culture positive for B. pseudomallei? | Thank you for the suggestion. Result on the blood culture report is added in line 139-140. The other detailed clinical investigation for this case have been published as part of a case series and is mentioned in the text (line no. 140-145) and reference is added (ref no. 19).  As such the focus of this investigation is the epidiological description and analytical case control study. |
|  | There is a very limited description of patient presentation: “The most common type was bacteremic melioidosis (53%).” This should be expanded, preferably in a table, to include: bacteremia, pneumonia, skin & soft tissue, organ involvement (i.e. liver abscess etc.), neuromelioidosis, no focus. | Thank you for the suggestion. The details on the type of presentations is added as below in line 158-160:  *Among 19 cases, the most common presentation was bacteremic melioidosis (58%), followed by skin and soft tissue (11%), septic arthritis (11%), pneumonia (5%), splenic abscess (5%), neuromelioidosis (5%) and no focus (5%).* |
|  | What was the difference between those with pneumonia vs no pneumonia in the context of the various environmental risk factors? It would be important to mention this information as inhalation is a common route of acquisition. | Thank you for this valuable suggestion. The following results are added (line no. 158-163):  *Out of 19 cases, five had lung involvement of which, one had focal lung involvement and four others had lung involvement with bacteremia. All five cases with pneumonia were exposed either to soil or stagnant water. Among the 14 non pneumonia cases, 13 had exposure to soil or stagnant water.* |
|  | What was the predominant presentation in the dry season compared to the wet season? | The predominant presentation was bloodstream infection, regardless of the season. |
|  | Table 1 should include the specific risk factors included in the results section. | We have included the results of all risk factors in table 1 as mentioned in result section. |
|  | Minor. | |
|  | Title: “August 2018” suggests that only this month was assessed. Consider rephrasing. | As suggested, we have removed “August” from the title. *The revised title is “ Risk factors for Melioidosis in Uduppi District, Karnataka, India, Januray 2017-July 2018* |
|  | Line 86: I assume the Gram stain revealed bipolar Gram negative staining? | Yes, clarified on line 63. |
|  | Line 88: incubated in room air/aerobic conditions or in 5% CO2? | The sentence is revised as *incubation at 37°C with 5% CO_2_* |
|  | Line 93: reference | Thank you. The reference has been added (ref no. 18) |
|  | Data analysis section: consider removing “we” | The paragraph has been rewritten removing “we”. Changes at line 121-124. |
|  | Line 133: transferred to intensive care | Thank you. The change is incorporated at line 134. |
|  | Line 139 – 141: suggest rewrite | The contrast MRI results are rewritten at line no. 140-143. |
|  | Line 154: neuromeliodosis should not be counted as a chronic comorbidity | Thank you. The text has been amended, removing neuromeliodosis as chronic comorbidity (line no. 156-158). |
|  | Line 154: “the most common type” suggest rewriting this sentence. | The sentence has been rewritten (line 158). |
|  | Line 165: “and having” with | The sentence has been rewritten (line 177). |
|  | Line 205: this sentence is misleading as there is no definition of fever of unknown origin mentioned in this manuscript. In this context it is more likely that the disease is either misdiagnosed or undiagnosed, and not a fever of unknown origin (e.g. a fever that lasts for >3 weeks or failure to reach a diagnosis after 1 week of appropriate investigations) | Fever of unknown origin has been deleted (line 219) |
|  | Line 206 – 209: grammar | The sentence has been rewritten. |
|  | Figure 2: it would be great if rainfall data could be included in this figure to demonstrate the correlation with the wet season. | Thank you for the suggestion. Included in figure 2. |

|  | Reviewer 2 |  |
| --- | --- | --- |
| S. No. | Comment | Response |
| 1 | Point 1: Line 202: “However, we tried to ensure comparability of exposures by enrolling controls during the rainy season, a time of high risk” It is not clear why authors tried to ensure comparability of exposure. Why were the controls not distributed throughout the year, as it would have brought out the seasonal risk factor, and risk factors associated with exposure better. Authors should not have tried to match the exposure. | Thank you for the comment. It is clarified that the investigators did not match cases and controls on the exposures (H/o contact with stagnant water or soil etc).  As more than half of the cases were identified in the rainy season, the intention of enrolling controls in the rainy season was to ensure comparability for risk between cases and control.  The text has been rephrased for clarity (line no. 214-215) as below:  *However, we ensured comparability of risk by enrolling controls during the rainy season, as more than half of the cases were reported during this season.* |
| 2 | Point 2: Line 139-141: The MRI description is not accurate. It cannot be “right side” of the corticospinal tract. Authors are directed to quote the case report which describes the same case from the region elaborately with MRI pictures in a previous publication that was published by the clinicians involved in case management- Chatterjee A, Saravu K, Mukhopadhyay C, Chandran V. Neurological Melioidosis Presenting as Rhombencephalitis, Optic Neuritis, and Scalp Abscess with Meningitis: A Case Series from Southern India. Neurol India. 2021 Mar-Apr;69(2):480-482. doi: 10.4103/0028-3886.314590. PMID: 33904481 | Thank you for the comment.  The contrast MRI results are rewritten at line no. 140-144 and the reference is added (ref no. 19) |
|  | Minor Points |  |
| 3 | Line 65-66: In India, sporadic case reports of melioidosis have been mostly from Karnataka and Tamil Nadu due to better diagnostic capacity in these regions. Authors fail to quote this paper which has for the first time described a case series of 25 patients from the same region as study setting, which has also described seasonal preponderance. Saravu K, Mukhopadhyay C, Vishwanath S, Valsalan R, Docherla M, Vandana KE, Shastry BA, Bairy I, Rao SP. Melioidosis in southern India: epidemiological and clinical profile. Southeast Asian J Trop Med Public Health. 2010 Mar;41(2):401-9. PMID: 20578524. | Thank you for this important suggestion. The text has been edited (line 65-67) and the reference added (ref no. 14-16)  The edited text is as follows:  *In India, cases of melioidosis have been recognized from different regions, however case identification is confined to few tertiary care centres.* |
| 4 | Line 154: Why is neuromeliodosis written as “other chronic co morbidity”? It is the very subject of investigation. | Thank you. The text has been amended, removing neuromeliodosis as chronic comorbidity (line no. 156-158). |
| 5 | Grammar and Typos are to be corrected in the manuscript Line 184. A systematic review from India for the period 1991-2018 found diabetes mellitus (to be ) a major predisposing condition in 70% of reported cases | Thank you. Correction made as per suggestion, line 194-195. |
| 6 | Line 193-194 “children and adolescents have poor outcomes, frequently resulting in either death or neurological impairment (37%) (5, 25)”. What is the possible explanation for this finding? | The possible explanation for this may be severe sepsis and its complications resulting from delay in treatment. This has been added at line 207. |
| 7 | Line 315: Muthusamy KA, Waran V, Puthucheary SD. Spectra of central nervous system melioidosis. J Clin 316 Neurosci. 2007;14:1213-5. A very old sole reference (2007) has been quoted while there are newer reports of neuromeliodosis.  Newer references and unique presentations that are reported recently(2015 and 2021) may be included for neuromeliodosis. 1.Chatterjee A, Saravu K, Mukhopadhyay C, Chandran V. Neurological Melioidosis Presenting as Rhombencephalitis, Optic Neuritis, and Scalp Abscess with Meningitis: A Case Series from Southern India. Neurol India. 2021 Mar-Apr;69(2):480-482. doi: 10.4103/0028-3886.314590. PMID: 33904481 2. Saravu K, Kadavigere R, Shastry AB, Pai R, Mukhopadhyay C. Neurologic melioidosis presented as encephalomyelitis and subdural collection in two male labourers in India. J Infect Dev Ctries. 2015 Nov 30;9(11):1289-93. doi: 10.3855/jidc.6586. PMID: 26623640. | Thank you for the suggestion, these references are added (ref no. 19 and 26) |

Reviewer 3

| S. No. | Comment | Response |
| --- | --- | --- |
| 1 | 1) Page 5 - Abstract. - The methodology seems to be misleading as only 19 cases underwent logistic matched case-control analysis. Yet, you have stated that 50 cases were identified. If I am not wrong, this maybe the incident cases for the retrospective epidemiological survey, all of which were not included in the statistical analysis. Please consider clarifying how many cases underwent the specified analysis. | Thank you for pointing this out. The 19 cases in the case control study were a subset of the 50 cases that were identified using the case definition from Jan 2013- July 2018. To minimize recall bias in the case control study, the most recent cases (Jan 2017-July 2018) were enrolled. This information is provided in the text at line 110-111.  This is now also added in the abstract for better clarity (line 6-8) as below:  *For the case control study, we enrolled a subset of cases admitted to hospital A from January 2017- July 2018. A control was resident of Udupi district admitted to hospital A in July (rainy season) 2018 with a non-infectious condition.*  A flowchart for enrollment is also added in appendix page 1. |
| 2 | Page 6 , Introduction - Line 64-66. I have to disagree with the statement that melioidosis reporting was limited to Karnataka and Tamil Nadu due to diagnostic limitations in other south-indian states in general. I believe diagnostic limitation may instead be tertiary care centres, that most south indian states do feature albeit far and between and due to the general unawareness of the condition. Citations no. 14 and 15 are quite outdated. I recommend citing more recent published reports from other states such as <https://doi.org/10.1155/2021/8154810>, <https://doi.org/10.3390/idr12030011> or even our published paper from <https://doi.org/10.1155/2021/8154810> | Thank you for the comment. As suggested, text has been modified in line 65-67 as under:  *In India, cases of melioidosis have been recognized from different regions, however case identification is confined to r few tertiary care centres.*  Newer suggested references have been added (ref no. 14,15). |
| 3 | Page 9 - Results - I - Case investigation - It would have been "nice" to have a MRI image of the patient with neuro-melioidosis - Completely optional but can improve reader engagement. | Thank you for this suggestion. The detailed clinical investigation for this case has been reported as part of a case series by the treating physicians with the MRI image and is referenced (ref no. 19).  As such the focus of this investigation is the analytical case control study. |
| 4 | Page 10 - Descriptive epidemiology - Only minimal baselines information is given on the 50 cases identified from 2013 to 2018. It would have been helpful to delve deep into the clinical presentation and cause of mortality of these patients ( and not just the 19), as data is probably already available from hospital record. Since objective evidence is available, I doubt recall bias would have limited the descriptive analysis. | Thank you for the suggestion. Clinical data was available for patients attending the hospital after 2016. The details on the type of presentations in 19 cases is added as below in line 134-136:  *Among 19 cases, the most common presentation was bacteremic melioidosis (58%), followed by skin and soft tissue (11%), septic arthritis (11%), pneumonia (5%), splenic abscess (5%), neuromelioidosis (5%) and no focus (5%).*  Additional following results are also added (line no. 160-163):  *Out of 19 cases, five had lung involvement of which, one had focal lung involvement and four others had lung involvement with bacteremia. All five cases with pneumonia were exposed either to soil or stagnant water. Among the 14 non pneumonia cases, 13 had exposure to soil or sta.gnant water.* |
| 5 | Page 11 - III- Case control study - Since there was no minimum sample size calculation, I am doubtful of the statistical power the regression analysis contains due to the relatively small sample analysed (wide confidence inferences noted for risk factors, many with statistical non-significance at alpha of 0.05). I recommend adding the power analysis and goodness-of-fit report, as it may inform the readers on the strength of the inferences made as the effect sizes (mOR) are quite large. | We have added the p-value results by conditional likelihood estimates in table 1for each of the analysis |
| 6 | The discussion is well drafted with a succinct comparison with existing literature, pragmatic suggestions to policy makers, with strengths and weaknesses of the study described in detail. | Thank you |
