# Supplementary material for: Risk factors for melioidosis in Udupi District, Karnataka, India, January 2017-July 2018
Source: PLOS Glob Public Health. 2022 Dec 13;2(12):e0000865. doi: 10.1371/journal.pgph.0000865 (PMC10021467; doi:10.1371/journal.pgph.0000865)
Supplement: Attachment — Submitted filename: Reviewer comments.docx [file pgph.0000865.s003.docx]

Reviewer #2: The authors have addressed reviewers comments satisfactorily

Reviewer #4: Reviewer comments

| Srl no. | Comment | Author Response |
| --- | --- | --- |
| Major | | |
| 1 | I think the article reads well after the first round of revisions. I do not have any major comments! | Thank you |
| 2 | Flow diagram in appendix is good. |  |
| Minor | | |
| 1 | Line 10: change title to “January 2017-July 2018” (rather than August 2018). | Line 10 revised.  The amended title is: Risk factors for Melioidosis in Udupi District, Karnataka, India, January 2017-July 2018 |
| 2 | Line 52: “in the tropics” | Line 52 revised |
| 3 | Line 62: Tsunamis | Line 62 revised |
| 4 | Line 66-67: Why is this the case? Is it because laboratory diagnosis is limited in other centres? | Clarification and additional information provided. The amended text reads as: *however case identification is confined to few tertiary centres due to limited diagnostic facilities (*line 66-67) |
| 5 | Line 77-78: Suggest rewording along lines of “identify risk factors to inform the initial public health response”. | Line 77-78 revised. |
| 6 | Line 86: “automated system” – which system? BacT/ALERT as per CSF or different? | Additional information proved (line 86). |
| 7 | Line 91: I think type 3 secretion system should be abbreviated: TTSS or T3SS not TTSI as brackets. | Line 91 revised. |
| 8 | Line 186-187: How significant was this association? Worth putting in the text? | The statement is revised. The amended statement is: we also found that sustaining cut injuries was an independent predictor in multivariate analysis. Melioidosis cases were six times more likely to be exposed to cut injuries compared to controls. (line 188-189). |
| 9 | Line 190: Suggest removing the word bacteria and just having “aerosolized B. pseudomallei”. | Revised as per suggestion at line 193. |
| 10 | Line 218: “We recommend sensitizing medical doctors..”- while I understand what you mean by this, it reads like a medical procedure! Would suggest something along the lines of “We recommend increased education of medical..” | Revised as per suggestion at line 221-222. |
| 11 | Table 1, line 370: Multiple chronic comorbidities is a listed risk factor, however is this 2 or more comorbidities? How was this defined? I think this should be defined better including potentially listing (as an endnote) what you called a comorbidity. | Revises as suggested in Table 1. |
